# Quantifying Facial Feminization Surgery’s Impact: Focus on Patient Facial Satisfaction

**DOI:** 10.1097/GOX.0000000000005366

**Published:** 2023-11-03

**Authors:** David P. Alper, Mariana N. Almeida, Kevin G. Hu, Heloise M. De Baun, Helia Hosseini, Mica C.G. Williams, Andrew Salib, Jinesh Shah, John A. Persing, Michael Alperovich

**Affiliations:** From * Division of Plastic Surgery, Department of Surgery, Yale School of Medicine, New Haven, Conn.; † Renaissance School of Medicine, Stony Brook University, Stony Brook, New York, N.Y.

## Abstract

**Background:**

Facial feminization surgery (FFS) has been associated with improving gender dysphoria in transgender patients. This study aimed to quantify the impact of surgery on patient facial satisfaction, using the FACE-Q and a quality-of-life (QoL) survey.

**Methods:**

Transgender female patients were recruited to complete the FACE-Q and the World Health Organization’s QoL Scale-Short Form (WHOQOL-BREF) if they were planning to or had undergone FFS at our institution. FACE-Q modules completed included “Satisfaction with Facial Appearance Overall,” individual facial attributes (forehead/eyebrows, nose, cheeks, cheekbone, chin, jawline, and neck), and the WHOQOL-BREF, which assesses patient QoL through four domains (physical, psychological, social relations, and environment). Both matched and unmatched analyses of preoperative versus postoperative cohorts were performed.

**Results:**

Overall, 48 patients participated in our study and completed 31 FACE-Q surveys preoperatively and 37 postoperatively. On average, patients were 37.2 ± 12.5 years old. FACE-Q scores increased significantly for all facial attributes and for Satisfaction with Facial Appearance Overall between cohorts (*P* < 0.05). The facial attribute with the greatest increase in satisfaction was the jawline, followed by the nose. The WHOQOL-BREF’s psychological and physical domains both improved significantly (*P* < 0.05). Wait time for surgery of less than 6 months (b = 22.42, *P* = 0.02) was associated with higher overall facial satisfaction, whereas age at surgery (b = −1.04, *P* < 0.01) was associated with lower overall facial satisfaction.

**Conclusions:**

Transgender female patients experienced significant improvements in facial satisfaction and QoL after FFS. Undergoing surgery at a younger age and shorter wait times for surgery were associated with increased overall facial satisfaction.

**Takeaways**

**Question:** How does facial feminization surgery (FFS) impact transgender female patients’ facial satisfaction?

**Findings:** Patients report improved facial satisfaction and quality of life after receiving FFS, particularly with respect to the nose, forehead, and chin. Facial satisfaction after surgery decreased with increasing patient age at surgery and increased with shorter wait times to surgery. Improvement in facial satisfaction occurs within six months postoperatively.

**Meaning:** FFS significantly improves facial satisfaction and quality of life for transgender female patients, but its benefits may be mitigated by older age at surgery and long wait times for surgery.

## INTRODUCTION

Facial feminization surgery (FFS) is a set of soft tissue and bone remodeling procedures that ideally confers a more “feminine” appearance. The procedures encompass the upper, middle, and lower thirds of the face, including frontal sinus setback, brow contouring, blepharoplasty, hairline advancement, rhinoplasty, mandibular shave, genioplasty, chondrolaryngoplasty, and fat grafting.^1–3^

FFS is a gender-affirming surgery (GAS) and is believed to significantly improve quality of life (QoL) for transgender patients.^4–7^ A recent study describes that transgender patients who underwent FFS experience a significantly greater public and self-perceived sense of femininity.^7^ In addition, these patients also reported decreased limitations in terms of social and professional activities.^7^ FFS has been shown to improve mental health outcomes, with transgender patients reporting significantly reduced rates of anxiety, depression, anger, and social isolation.^5^

Although FFS plays an important role in gender-affirmation, there remains limited accuracy and heterogenous methods of reporting the quality of surgical outcomes related to patient satisfaction of facial appearance.^8,9^ The FACE-Q instrument is a validated patient-reported outcome measure (PROM) that has become increasingly widespread in clinical and research practices to assess facial outcomes.^10^

The FACE-Q has been previously used to assess self-measured satisfaction with facial appearance for transgender women who have not undergone FFS.^11^ Although some preliminary studies have applied the FACE-Q to transgender patients,^12–14^ no studies have directly evaluated facial satisfaction before and after receiving expanded gender-affirming facial surgery. Though Morrison et al conducted a thorough analysis of pre- and postprocedure QoL measures, they only assess patient face satisfaction after FFS at two time points and not before FFS.^6^ With an increasing demand for FFS, evaluation of preoperative and postoperative outcomes can offer direct evidence-based practices about the impact of surgery on patient satisfaction and assist in setting patient expectations. In this study, we used the FACE-Q to quantify the impact of FFS on patients’ facial satisfaction preoperatively and postoperatively.

## METHODS

### Patient Population

Institutional review board approval was obtained before study initiation (HIC# 2000031685), and patients were consented before participation. Patients were recruited if they had undergone or were going to receive initial FFS. Patients were surveyed about their experiences relative to only their initial surgery. Patient gender was not explicitly queried with the two-step method, but all patients had been assigned male sex at birth and identified as transgender female during the course of preoperative evaluations.^15^ Although the World Professional Association for Transgender Health does not provide specific criteria before performing FFS, at our institution, patients are required to have persistent, well-documented gender dysphoria; capacity for informed consent and decision-making; age over 18; and the long-term support of a therapist, endocrinologist, or other healthcare professional before receiving FFS.^16^ Patients are strongly encouraged to wait until after completion of 1 year of hormone replacement therapy and at least 1 year of living in their congruent gender role. The procedures may include frontal sinus setback/forehead contouring, rhinoplasty, genioplasty, mandibular contouring, chondrolaryngoplasty, brow lift, hairline advancement, and fat grafting. Patients completed the FACE-Q, World Health Organization’s QoL Scale-Short Form (WHOQOL-BREF), and a demographics survey.

### FACE-Q

The FACE-Q is a PROM composed of over 40 independently functioning scales that measure satisfaction of patients undergoing facial procedures. Use of this questionnaire, authored by Drs. Klassen, Pusic, and Cano, was permitted under license from Memorial Sloan Kettering Cancer Center, New York, USA.^17,18^ Patients were asked preoperatively and postoperatively to complete the following FACE-Q scales: “Satisfaction with Facial Appearance Overall,” “Satisfaction with Facial Structure” (specific segments of the face were forehead/eyebrows, nose, cheeks, cheekbone, chin, jawline, and neck), and “Psychological Function.” With facial appearance in mind, the Psychological Function scale measures concepts, such as happiness, confidence, and self-acceptance (eg, “I like myself,” “I feel happy,” “I am accepting of myself”). Patients postoperatively completed the modules “Satisfaction with Decision” and “Satisfaction with Outcomes.” Each question consisted of a four-point Likert scale from 1 (very dissatisfied/definitely disagree) to 4 (very satisfied/definitely agree). The modules were scored individually, and Rasch-transformed scores (range 0–100) were calculated from raw scores.

### WHOQOL-BREF

The WHOQOL-BREF was used to assess patient QoL preoperatively and postoperatively. The survey covers four domains: physical, psychological, social relationships, and environment. The obtained raw scores were converted to domain scores on a scale from 0 to 100, with higher scores denoting higher QoL.

### Demographics Survey

A demographics survey was used to assess patients’ experiences obtaining GAS. Questions included time spent living openly in their desired gender, time spent waiting for FFS, prior GAS, and education level.

### Statistical Analysis

Continuous variables were summarized using means and standard deviations, whereas categorical variables were expressed as proportions. The mean FACE-Q and QoL scores were compared between the preoperative and postoperative cohorts, using unpaired *t* test for continuous variables and *χ*2 test for categorical variables. Scores were also compared by the number and types of procedures. Patients who completed the FACE-Q and QoL modules both preoperatively and postoperatively (matched cohort) were completed using paired *t* tests. Univariate linear regression was used to determine the relationship between satisfaction with facial appearance, overall scores, and patient demographics. Analysis of variance was used to compare postoperative facial satisfaction scores between patients who completed the FACE-Q at 6 months, 6–12 months, 12–18 months, 18–24 months, and more than 24 months postoperatively. Significance was set at *P* less than 0.05, and all statistical analysis was completed in SAS 9.4.

## RESULTS

### Patient Characteristics

Of the patients recruited, 82.8% (48/58) completed the FACE-Q preoperatively, postoperatively, or both. Patients who did not complete the FACE-Q at any point did not differ from patients who did complete the FACE-Q with respect to age at surgery, number of procedures performed, procedure types, and proportion who reported dissatisfaction. (**See table, Supplemental Digital Content 1**, which displays the comparison of survey responders to survey nonresponders. http://links.lww.com/PRSGO/C834.) One patient who completed the FACE-Q preoperatively was lost to follow-up. All remaining patients recruited preoperatively completed the FACE-Q postoperatively. Overall, the FACE-Q was completed a total of 68 times: 31 preoperatively and 37 postoperatively (Table 1). Twenty patients completed the FACE-Q both preoperatively and postoperatively (matched cohort). On average, patients were 37.2 ± 12.5 years old, ranging from 19.7 to 73.8 years old. Among patients that only completed the FACE-Q postoperatively, the mean time from surgery to completion was 15.0 ± 14.5 months (range: 5.0–61.6), whereas the matched cohort’s mean time was 6.9 ± 2.2 months (range: 5.0–14.7). The most common procedure was frontal sinus setback/brow contouring (83.3%, n = 40) followed by genioplasty (81.3%, n = 38). The majority of patients received procedures on at least two regions of the face. (**See table, Supplemental Digital Content 2**, which displays the tabulation of procedure types and the combinations of facial regions operated on. http://links.lww.com/PRSGO/C835.)

**Table 1.** Patient Demographics

Characteristics	Value (%)
No. patients	48
FACE-Q completions	
Total	68
Preoperatively	31 (45.6)
Postoperatively	37 (54.4)
Mean time from surgery to FACE-Q completion, mo (SD)	15.0 ± 14.5
Matched preoperatively and postoperatively	20
Mean time from FGAS to FACE-Q completion, mo (SD)	6.9 ± 2.2
Mean age at surgery, yr. ± SD	37.2 ± 12.5
Race	
White, non-Hispanic	31 (64.6)
Non-White	17 (35.4)
Average number of procedures (SD)	5.9 ± 2.0
Procedure type	
Frontal sinus setback/brow contouring	40 (83.3)
Rhinoplasty	38 (79.2)
Genioplasty	39 (81.3)
Mandibular contouring	36 (75.0)
Fat grafting	39 (81.3)
Chondrolaryngoplasty	18 (37.5)
Hairline advancement	35 (72.9)
Brow lift	37 (77.1)
Time spent living openly in gender	
<1 year	0 (0.0)
1–3 years	12 (25.0)
3–5 years	11 (22.9)
5–10 years	8 (16.7)
10–20 years	9 (18.8)
>20 years	3 (6.3)
Missing	5 (10.4)
Time spent waiting for FGAS	
<6 months	9 (18.8)
6–12 months	15 (31.3)
12–18 months	4 (8.3)
18–24 months	4 (8.3)
2–3 years	5 (10.4)
3–4 years	1 (2.1)
4–5 years	3 (6.3)
>5 years	3 (6.3)
Missing	4 (8.3)
Received other GAS	
Top surgery	17 (35.4)
Bottom surgery	6 (12.5)
Top and bottom surgery	2 (4.2)
None	16 (33.3)
Prefer not to answer	3 (6.3)
Missing	4 (8.3)
Highest level of education completed	
No high school	1 (2.1)
Some high school	2 (4.2)
High school graduate or equivalent	9 (18.8)
Some college/technical school/associate’s degree	7 (14.6)
College graduate	10 (20.8)
Graduate school	4 (8.3)
Missing	15 (31.3)

The waiting time before receiving FFS was more than 2 years in 25% (12/48) of patients and more than 4 years in 12.5% (6/48) of patients. Before FFS, 33.3% (16/48) of patients had not received previous GAS, whereas 52.1% (25/48) had received a combination of top surgery, bottom surgery, or both. Notably, the preoperative and postoperative cohorts were similar in age, race, and procedures received (Table 2).

**Table 2.** Preoperative versus Postoperative Patient Demographics

Characteristic	Preoperative, Value (%)	Postoperative, Value (%)	*P*
No. of patients	31	37	
Mean age at surgery, y (SD)	36.0 (11.4)	38.6 (13.7)	0.4
Race			
White non-Hispanic	22 (71.0)	27 (73.0)	0.86
Non-White	9 (29.0)	10 (27.0)	0.86
Procedure type			
Frontal sinus setback/brow contouring	29 (93.5)	31 (83.8)	0.22
Rhinoplasty	24 (77.4)	29 (78.4)	0.93
Genioplasty	27 (87.1)	30 (81.1)	0.51
Mandibular contouring	26 (83.9)	27 (73.0)	0.29
Fat grafting	28 (90.3)	29 (78.4)	0.33
Chondrolaryngoplasty	9 (29.0)	15 (40.5)	0.19
Hairline advancement	24 (77.4)	27 (73.0)	0.68
Brow lift	25 (80.6)	28 (75.7)	0.63

### FACE-Q and QoL Scores: Unmatched Cohort

Among all patients, the mean score for Satisfaction with Facial Appearance Overall increased by 31.0 (35.9 ± 13.2 preoperatively to 66.9 ± 21.6 postoperatively, *P* < 0.001). The mean FACE-Q scores for satisfaction for all individual facial attributes (forehead/eyebrows, nose, cheeks, cheekbone, chin, jawline, and neck) increased significantly preoperatively to postoperatively (Table 3). The facial region with the greatest increase was the jawline, followed by the nose and forehead/eyebrows. The mean score for the physical domain of the WHOQOL-BREF increased by 20.4 (46.0 ± 14.1 preoperatively to 66.4 ± 25.5 postoperatively, *P* < 0.001). The mean score for the psychological domain of the WHOQOL-BREF increased by 21.0 (45.8 ± 22.5 preoperatively to 66.8 ± 25.7 postoperatively, *P* < 0.001). The mean score for social relationships and the environmental domain of WHOQOL-BREF did not increase significantly.

**Table 3.** Mean FACE-Q and QoL Scores Preoperatively and Postoperatively

Characteristics	Preoperative	Postoperative	Change	*P*
FACE-Q scale (range, 0-100)				
Satisfaction with facial appearance overall	35.9 (13.2)	66.9 (21.6)	31.0	**<0.001**
Forehead/eyebrows	43.5 (23.0)	81.5 (18.2)	38.0	**<0.001**
Nose	40.9 (20.8)	79.4 (19.0)	38.5	**<0.001**
Cheeks	47.7 (23.5)	78.2 (21.4)	30.5	**<0.001**
Cheekbones	52.1 (28.1)	82.6 (18.5)	30.5	**<0.001**
Chin	36.3 (21.8)	69.4 (28.7)	33.1	**<0.001**
Jawline	36.3 (25.0)	75.0 (22.5)	38.7	**<0.001**
Neck	56.3 (23.1)	72.7 (27.0)	16.4	**0.01**
Psychological function	40.3 (24.8)	69.4 (26.9)	29.1	**<0.001**
Satisfaction with decision	—	78.5 (28.8)	—	—
Satisfaction with outcomes	—	70.1 (30.3)	—	—
WHOQOL-BREF (range, 0-100)				
Physical	46.0 (14.1)	66.4 (25.5)	20.4	**<0.001**
Psychological	45.8 (22.5)	66.8 (25.7)	21.0	**<0.001**
Social relationships	59.4 (26.7)	62.6 (23.8)	3.2	0.62
Environmental	59.8 (18.5)	63.7 (16.1)	3.9	0.37

*P* values in bold indicate statistical significance of *P*<0.05.

### FACE-Q and QoL Scores: Matched Cohort

**Fig. 1.**
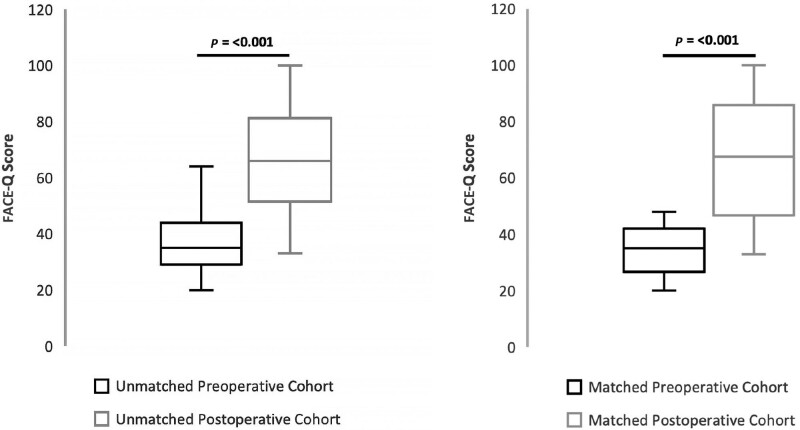
FACE-Q satisfaction with facial appearance overall in unmatched and matched cohorts.

Among the matched cohort of patients who completed the FACE-Q both preoperatively and postoperatively (Table 4), FACE-Q scores increased significantly in all scales. Patients’ Satisfaction with Facial Appearance Overall scores were similar to those of the unmatched cohort (Fig. 1). The facial region with the greatest increase was the jawline followed by forehead/eyebrows and nose. The mean score for the physical domain of the WHOQOL-BREF increased by 14.3 (46.8 ± 22.5 preoperatively to 59.8 ± 21.6 postoperatively, *P* < 0.001). The mean score for the psychological domain of the WHOQOL-BREF increased by 14.0 (45.8 ± 22.5 preoperatively to 59.8 ± 21.6 postoperatively, *P* < 0.001). The mean score for social relationships and the environmental domain of the WHOQOL-BREF did not increase significantly.

**Table 4.** Mean FACE-Q and QoL Scores Matched Cohort

Characteristics	Preoperative	Postoperative	Change	*P*
**FACE-Q scale (range, 0-100**)				
Satisfaction with facial appearance overall	33.5 (11.6)	66.8 (22.5)	33.3	**<0.001**
Forehead/eyebrows	39.2 (21.3)	82.2 (20.0)	43.0	**<0.001**
Nose	40.2 (24.9)	79.5 (20.3)	39.3	**<0.001**
Cheeks	43.2 (21.9)	74.0 (21.0)	30.8	**<0.001**
Cheekbones	45.1 (28.00)	82.7 (19.0)	37.6	**<0.001**
Chin	35.2 (14.7)	70.2 (24.6)	35.0	**<0.001**
Jawline	30.5 (17.8)	75.5 (21.2)	45.0	**<0.001**
Neck	51.1 (24.2)	69.0 (27.2)	17.9	**0.01**
Psychological function	38.0 (19.7)	64.9 (25.8)	26.9	**<0.001**
Satisfaction with decision	—	81.9 (21.6)	—	—
Satisfaction with outcomes	—	70.3 (28.3)	—	—
**WHOQOL-BREF (range, 0-100**)				
Physical	46.4 (13.7)	60.7 (17.9)	14.3	**0.001**
Psychological	45.8 (23.4)	59.8 (21.6)	14.0	**<0.001**
Social relationships	60.0 (25.5)	61.7 (25.0)	1.7	0.66
Environmental	60.1 (17.0)	63.0 (17.9)	2.9	0.28

*P* values in bold indicate statistical significance of *P*<0.05.

### Change in FACE-Q Score by Procedure and Univariate Analysis

Changes in individual FACE-Q scales (eg, Satisfaction with Facial Attributes: Nose) preoperatively to postoperatively did not reach significance when compared by anatomic region between patients who underwent the procedure in that region versus those who did not. Univariate analysis showed that age at surgery and less than 6 months of wait time to receive surgery were each independently associated with multiple FACE-Q subscales (Table 5). Increasing age at surgery was associated with lower satisfaction in Overall Facial Appearance (b = −1.04, *P* < 0.001), Satisfaction with Decision (b = −1.13, *P* = 0.01), and Satisfaction with Outcomes (b = −1.12, *P* = 0.01). Less than 6 months of wait time to receive surgery was positively associated with higher satisfaction in Overall Facial Appearance (b = 22.42, *P* = 0.02), Satisfaction with Decision (b = 26.5, *P* < 0.05), Satisfaction with Outcomes (b = 34.33, *P* = 0.01), and Psychological Function (b = 26.63, *P* = 0.02).

**Table 5.** Univariate Analysis Evaluating the Association between Wait Times and Age at Surgery with Postoperative FACE-Q Facial Satisfaction Scores

	Satisfaction with Overall Facial Appearance			Satisfaction with Decision			Satisfaction with Outcomes			Psychological Function		
	b	95% CI	*P*	b	95% CI	*P*	b	95% CI	*P*	b	95% CI	*P*
Age at surgery (y)	-1.04	-1.49, -0.58	**<0.001**	-1.13	-1.89, -0.37	**0.01**	-1.12	-1.89, -0.36	**0.01**	-0.65	-1.33, 0.03	0.06
Less than 6 months wait time to receive surgery	22.42	4.72, 40.11	**0.02**	26.5	0.04, 52.96	**0.05**	34.33	9.07, 59.59	**0.01**	26.63	5.48, 47.77	**0.02**

*P* values in bold indicate statistical significance of *P*<0.05.

On analysis of variance, there was no difference in FACE-Q Satisfaction with Facial Appearance Overall scores between patients who completed the survey at 6 months, 6–12 months, 12–18 months, 18–24 months, and 24–48 months postoperatively (*P* = 0.90).

## DISCUSSION

The goal of FFS is to achieve an external facial appearance concordant with the patient’s perceived gender. When comparing preoperative and postoperative cohorts, patients reported significant increases in scores in the psychological and physical QoL domains, as measured by the WHOQOL-BREF. Additionally, patients reported significant increases in satisfaction with facial appearance overall and individual facial attributes as measured by the FACE-Q. The greatest improvement was seen in the jawline, followed by the nose and forehead/eyebrow. When these variables were assessed in the matched cohort, which includes patients who completed the survey both preoperatively and postoperatively, we found consistent results demonstrating that FFS improves facial satisfaction and QoL for transgender women.

Analyzing individual FACE-Q scales (eg, Satisfaction with Facial Attributes: Nose) in patients who underwent a procedure to alter a specific facial attribute (eg, rhinoplasty for nose) revealed that patients who received rhinoplasty, genioplasty, and mandibular contouring generally reported higher satisfaction related to the revised facial structure than those who did not (Table 6). This higher level of satisfaction was not statistically significant, potentially due to sample size. Most patients underwent multiple procedures simultaneously (**Supplemental Digital Content 2**, http://links.lww.com/PRSGO/C835).

**Table 6.** Mean Change in FACE-Q Scores between Patients Who Obtained Specific Procedures

	Had the Procedure	Did Not Have the Procedure	*P*
Frontal sinus setback/brow contouring			
No. patients	20	0	
Change in “satisfaction with facial attributes: forehead/eyebrows” (SD)	+43.0 (24.9)	—	—
Rhinoplasty			
No. patients	15	5	
Change in “satisfaction with facial attributes: nose” (SD)	+43.9 (20.8)	+25.4 (36.3)	0.17
Genioplasty			
No. patients	17	3	
Change in “satisfaction with facial attributes: chin” (SD)	+37.6 (22.7)	+20.3 (35.2)	0.27
Mandibular contouring			
No. patients	17	3	
Change in “satisfaction with facial attributes: jawline” (SD)	+49.5 (20.4)	+19.3 (41.6)	0.057
Chondrolaryngoplasty			
No. patients	6	14	
Change in “satisfaction with facial attributes: neck” (SD)	+16.3 (37.4)	+18.5 (25.4)	0.88

Among the postoperative cohort, analysis of variance found no significant differences between patients who completed the FACE-Q at different time periods, suggesting that there was no decrease in facial satisfaction over time and that FFS provides a lasting impact.

In addition to increased satisfaction with facial appearance, patients reported significant increases in scores in the psychological and physical QoL domains as measured by the WHOQOL-BREF. These findings are consistent with other studies demonstrating the ability of FFS to improve QoL through multiple psychosocial domains.^5,8,19^ In this study, however, social relationships and the environmental domain of the QoL questionnaire did not demonstrate statistically significant increases, possibly indicating that the immediate effects following FFS are more intrinsic in nature. Extrinsic factors such as interpersonal relationships and surroundings may take longer to show significant improvement. This differed from the findings of Caprini et al, which showed positive effects in social relations through improvements in social isolation after FFS.^5^

Patient-related factors that were associated with more favorable outcomes in our cohort by means of univariate analysis included younger age at surgery and a wait time of less than 6 months. Both factors were associated with higher scores for satisfaction in facial appearance overall and satisfaction with decisions. The impact of patient age on satisfaction may be due to age-related differences in soft-tissue structure and flexibility, different degrees of support and acceptance among the patient’s peer group, or even generational differences in aesthetic preferences. Age has been found to be a predictor of patient satisfaction in an international multicenter study evaluating outcomes of 66 patients receiving FFS.^6^ These findings underscore the importance of making FFS more accessible and affordable for transgender women, as prolonged wait times and an older age at surgery may result from obstacles related to accessibility and insurance coverage.

Existing literature has suggested positive satisfaction with FFS, but most studies have used general QoL surveys rather than surveys specific to facial appearance.^19^ In addition, there remain nonuniversal and heterogenous methods for assessing PROMs in this patient population.^8,9^ Unfortunately, there are no widely accepted or validated methods. However, the FACE-Q is being used increasingly in studies to assess facial satisfaction in transgender populations that have received gender-affirming treatment.^11–14^

Alcon et al used the FACE-Q to assess facial satisfaction of 17 transgender patients after single-stage FFS. They found high levels of satisfaction with outcomes and low levels of appearance-related psychosocial distress. However, that study lacked both a comparison group and preoperative assessment of patient satisfaction.^13^ In another study, Perrillat et al used the FACE-Q following upper third feminization but only assessed patients retrospectively. This group reported that 67.2% were satisfied with the outcome, and 79% were satisfied or very satisfied with the position of their eyebrows postoperatively.^14^ Our cohort had comparably high levels in satisfaction with the outcome and overall appearance of forehead/eyebrows postoperatively.

Similarly, another recent study used the FACE-Q to assess upper third feminization using custom bone section guides in patients who underwent frontoplasty and frontal sinus impaction osteotomies. Their study included a subanalysis of a smaller cohort (n = 15) who completed both the preoperative and postoperative questionnaires, and found significant increases in satisfaction with overall facial appearance and forehead/eyebrow appearance scales of the FACE-Q.^12^ Our study builds upon these pilot studies using the FACE-Q by evaluating patient satisfaction from full-FFS both preoperatively and postoperatively, which, to our knowledge, has never been reported.

Unfortunately, access to FFS in the United States has been inconsistent.^20^ Trends in regional and state-based differences in the number of FFS cases are unexplained by variations in population and suggest unequal access to care.^21,22^ In addition, top and bottom surgery are more frequently covered than facial feminization due to insurers categorizing FFS as an elective procedure.^20,23^ Despite this, the patients in this study were all covered by insurance, preventing further study of whether self-pay impacts patient satisfaction. Although Caprini et al did not examine self-pay, they found that patients with private insurance had worse anxiety and anger outcome scores than those with public insurance, suggesting that insurance type may meaningfully impact patient outcomes.^5^ It is worth noting the September 2022 update of World Professional Association for Transgender Health’s authoritative Standards of Care (SOC-8), reclassifying FFS as a medically necessary procedure.^16^ This recognition highlights the importance of FFS as an essential component of transgender healthcare. Transgender women cite limitations in expressing their desired gender due to difficulty masking masculine facial features such as the jawline.^24^ It is our opinion that there should be equal importance afforded to FFS relative to other GAS. Our cohort reflects this priority given that for one-third of patients, FFS was their first choice of GAS. Overall, the results of this study demonstrate increased satisfaction with facial appearance, improved mental health, and QoL, further emphasizing the importance of FFS for transgender women.

There are several limitations to this study that deserve consideration. First, although the FACE-Q has been utilized in numerous studies of transgender patients, it is not validated in this patient population and does not have trans-specific questions. The GENDER-Q is currently being calibrated to measure outcomes specific to gender-affirming treatments and could be used in future FFS studies.^25^ Secondly, small sample sizes limit the strength of our conclusions, such as the significance of trends in individual FACE-Q scales based on anatomic region operated on and the significance of the matched cohort analysis. At our institution, we typically perform full facial feminization, with surgery including procedures in the upper, middle, and lower thirds of the face. Small sample sizes also hamper multivariable regression power, resulting in the univariate regressions seen here. Consequently, there is a risk of shared variance between predictor variables. Further studies with more patients will allow a more nuanced understanding of not just how these factors impact patient outcomes but also how they interact with one another. Lastly, while we found no significant differences between patients who completed the FACE-Q at different time periods, our follow-up length was limited.

The study’s PROM methodology can also be improved. Although nonresponders and responders did not differ in demographics and procedural details, standardized PROM practices may increase responder yield in future studies.^26^ In this study, patients seemed to be more likely to start or complete a PROM when given accurate estimates of survey duration and details on how their participation may improve patient care. Additionally, a minimal important change threshold was not computed for either of the PROMs used in this study in the manner of Terwee et al.^27^ However, patients overall expressed significant satisfaction with their decision to receive FFS and satisfaction with their surgical outcomes (Tables 3 and 4). Future studies may benefit from the addition of anchoring questions to PROMs to better evaluate whether each patient received a significant clinical benefit from their choice of procedure.

## CONCLUSIONS

Transgender patients experienced significant improvements in satisfaction with facial appearance and QoL after FFS. Undergoing surgery at a younger age and shorter wait times before surgery were associated with increased overall facial satisfaction.

## DISCLOSURES

Dr. Michael Alperovich receives funding from CTSA (Grant Number KL2 TR001862) from the National Center for Advancing Translational Science, a component of the National Institutes of Health (NIH), and consults for Johnson & Johnson and LifeNet Health. Dr. Alperovich’s research was also partially funded by a Plastic Surgery Foundation Craniomaxillofacial Research Grant. All the other authors have no financial interest to declare in relation to the content of this article.

## Supplementary Material


