# Supplementary material for: Quantifying Facial Feminization Surgery’s Impact: Focus on Patient Facial Satisfaction
Source: Plast Reconstr Surg Glob Open. 2023 Nov 3;11(11):e5366. doi: 10.1097/GOX.0000000000005366 (PMC10624460; doi:10.1097/GOX.0000000000005366)
Supplement: Supplementary file 1 [file gox-11-e5366-s001.pdf]

SDC 1. Comparison of survey responders to survey non-responders

	Survey Responders (n=48)	Survey Non-Responders (n=10)	p-value
Mean age at surgery, yr. ± SD	37.2 ± 12.5	35.2 ± 10.45	0.77
Average number of procedures (SD)	5.9 ± 2.0	5.6 ± 1.6	0.69
**Procedure Type**			
Frontal Sinus Setback/Brow Contouring, n(%)	40 (83.3)	8 (80.0)	0.8
Rhinoplasty, n(%)	38 (79.2)	7 (70.0)	0.54
Genioplasty, n(%)	39 (81.3)	7 (70.0)	0.43
Mandibular Contouring, n(%)	36 (75.0)	9 (90.0)	0.31
Fat grafting, n(%)	39 (81.3)	7 (70.0)	0.2
Chondrolaryngoplasty, n(%)	18 (37.5)	6 (60.0)	0.43
Hairline advancement, n (%)	35 (72.9)	6 (60.0)	0.27
Brow lift, n (%)	37 (77.1)	6 (60.0)	0.42
Reported Dissatisfaction	4 (8.3)	1 (10.0)	0.28
