# Supplementary material for: Quantifying Facial Feminization Surgery’s Impact: Focus on Patient Facial Satisfaction
Source: Plast Reconstr Surg Glob Open. 2023 Nov 3;11(11):e5366. doi: 10.1097/GOX.0000000000005366 (PMC10624460; doi:10.1097/GOX.0000000000005366)
Supplement: Supplementary file 2 [file gox-11-e5366-s002.pdf]

SDC 2. Tabulation of procedure types and the combinations of facial regions operated on.

Face Region/Feature	Number of Patients (n = 48)
**Upper Face only**	4
Forehead	4
Brow	2
Eyes	3
Hairline	1
**Middle Face only**	4
Nose	4
**Lower Face only**	4
Jaw	0
Chin	1
Neck	4
**Exactly two regions**	13
Upper and Lower Face	4
Upper and Middle Face	5
Middle and Lower Face	4
**All three regions**	23
